# Loss of Resting-State Posterior Cingulate Flexibility Is Associated with Memory Disturbance in Left Temporal Lobe Epilepsy

**DOI:** 10.1371/journal.pone.0131209

**Published:** 2015-06-25

**Authors:** Linda Douw, Catherine L. Leveroni, Naoaki Tanaka, Britt C. Emerton, Andrew C. Cole, Claus Reinsberger, Steven M. Stufflebeam

**Affiliations:** 1 Department of Radiology, Athinoula A. Martinos Center for Biomedical Imaging, Massachusetts General Hospital, Charlestown, MA, United States of America; 2 Department of Radiology, Harvard Medical School, Boston, MA, United States of America; 3 Department of Anatomy and Neurosciences, VU University Medical Center, Amsterdam, The Netherlands; 4 Department of Psychiatry, Massachusetts General Hospital, Boston, MA, United States of America; 5 Department of Neurology, Massachusetts General Hospital, Boston, MA, United States of America; 6 Department of Neurology, Brigham and Women’s Hospital, Boston, MA, United States of America; 7 Institute of Sports Medicine, Faculty of Science, University of Paderborn, Paderborn, Germany; Institute of Psychology, Chinese Academy of Sciences, CHINA

## Abstract

The association between cognition and resting-state fMRI (rs-fMRI) has been the focus of many recent studies, most of which use stationary connectivity. The dynamics or flexibility of connectivity, however, may be seminal for understanding cognitive functioning. In temporal lobe epilepsy (TLE), stationary connectomic correlates of impaired memory have been reported mainly for the hippocampus and posterior cingulate cortex (PCC). We therefore investigate resting-state and task-based hippocampal and PCC flexibility in addition to stationary connectivity in left TLE (LTLE) patients. Sixteen LTLE patients were analyzed with respect to rs-fMRI and task-based fMRI (t-fMRI), and underwent clinical neuropsychological testing. Flexibility of connectivity was calculated using a sliding-window approach by determining the standard deviation of Fisher-transformed Pearson correlation coefficients over all windows. Stationary connectivity was also calculated. Disturbed memory was operationalized as having at least one memory subtest score equal to or below the 5^th^ percentile compared to normative data. Lower PCC flexibility, particularly in the contralateral (i.e. right) hemisphere, was found in memory-disturbed LTLE patients, who had up to 22% less flexible connectivity. No significant group differences were found with respect to hippocampal flexibility, stationary connectivity during both rs-fMRI and t-fMRI, or flexibility during t-fMRI. Contralateral resting-state PCC flexibility was able to classify all but one patient with respect to their memory status (94% accuracy). Flexibility of the PCC during rest relates to memory functioning in LTLE patients. Loss of flexible connectivity to the rest of the brain originating from the PCC, particularly contralateral to the seizure focus, is able to discern memory disturbed patients from their preserved counterparts. This study indicates that the dynamics of resting-state connectivity are associated with cognitive status of LTLE patients, rather than stationary connectivity.

## Introduction

Temporal lobe epilepsy (TLE) is the most commonly occurring form of focal epilepsy. Many patients suffer from cognitive impairments in general, many have memory deficits [1]. The functional imaging correlates of memory function in TLE, however, remain elusive. Resting-state fMRI (rs-fMRI) connectivity patterns have been explored, mostly focusing on the default mode network (DMN), which is the most consistently connected network during this no-task condition [2]. Several studies suggest that TLE is marked by a disconnection between the anterior and posterior parts of the DMN [3–8], in addition to altered ipsilateral and contralateral connectivity between DMN regions, such as the posterior cingulate cortex (PCC), precuneus, and hippocampus [7,9–12]. Decreased ipsilateral and increased contralateral functional connectivity of the hippocampus and/or PCC are generally related to better memory performance [9–12]. These regions are part of an interconnected network, with fibers running from the hippocampus to the PCC via the cingulum bundle.

The dynamical properties of resting-state DMN connectivity, however, have not been investigated with respect to memory functioning; the prior studies averaged the stationary resting-state connectivity matrices. Although this is desirable from a signal-to-noise standpoint and also pertains to classical stationary network topology, memory functioning may also depend heavily on dynamical functioning, or flexibility, of the brain network [13,14]. Recent studies have demonstrated a relationship between flexibility and cognitive functioning. Using task fMRI (t-fMRI), it has been shown that high flexibility of connections between different subsystems of the brain during a motor learning task is beneficial for performance on that task [15]. During the resting-state, a decreased number of alternating connectivity states is present in schizophrenia patients compared to healthy controls [16]. Furthermore, pre-stimulus flexibility of connectivity has been correlated with subsequent performance on a vigilance task [17]. Increased resting-state switching between several ‘brain states’ has also been found to correlate positively with a number of cognitive outcome measures [18]. In absence epilepsy, dynamic reconfiguration of connectivity, particularly of the PCC, has been reported going from a pre-ictal to ictal state [8]. However, the influence of resting-state flexibility on memory functioning has not been investigated, particularly in the setting of TLE.

We therefore aim to investigate the association between network flexibility of the ipsilateral and contralateral hippocampus and PCC with memory functioning in left TLE (LTLE) patients in a proof of concept study. We compare flexibility to stationary connectivity, both during resting-state and during a memory-encoding task. Our hypothesis is that high resting-state flexible connectivity is necessary for preserved out-of-scanner memory functioning, more so than stationary and task-based connectivity.

## Materials and Methods

### Ethics statement

This study was approved by the institutional review board of Massachusetts General Hospital, and was performed in accordance with the Declaration of Helsinki. All patients gave written informed consent before participation.

### Patient characteristics

LTLE patients were retrospectively analyzed after visits to the Massachusetts General Hospital (MGH) between December 2009 and April 2013. Patients were diagnosed with medically refractory epilepsy, and were referred for neuroimaging as part of a presurgical work-up. Inclusion criteria were (1) age > 18 years old, and (2) LTLE based on comprehensive presurgical evaluation including clinical history, semiology, and EEG video monitoring, in accordance with the epilepsy classification by the International League Against Epilepsy (ILAE), and (3) right-handedness. Exclusion criteria were (1) previous surgical intervention, and (2) neurological or psychiatric comorbidities. All patients underwent rs-fMRI, t-fMRI (semantic decision test [19]), and clinical neuropsychological testing. Clinical information (i.e. seizure frequency, anti-epileptic drug (AED) use, duration of disease, etcetera) was collected by medical chart review. Of the 16 patients included in the final sample (see Results section), 9 underwent left temporal lobe resection after this study; all were subsequently seizure free.

### Memory functioning

All patients were seen under supervision of a board-certified clinical neuropsychologist. The neuropsychological test battery administered per patient varied based on clinical background. Verbal memory was assessed using the Selective Reminding Test [20] or Rey Auditory Verbal Learning Test [21] and/or the Logical Memory subtest from the Wechsler Memory Scale, 3^rd^ Edition (WMS-III [22]). Visual memory was assessed using the 7/24 memory test [23], the Brief Visuospatial Memory Test-Revised [24] and/or the Faces subtest from the WMS-III.

A memory score was determined, by first converting all scores on memory tests to percentile scores based on normative data available for each subtest (corrected for age, gender and educational level). Memory disturbance was then defined as having a 5^th^ percentile score (i.e. 2 standard deviations, one-tailed) or lower compared to the normative scores on one or more test. This cut-off is commonly used in neuropsychological practice to determine clinical disturbance. Patients without scores above the 5^th^ percentile on all memory subtests were assumed to have preserved memory function.

### MRI acquisition

All MRI data were collected using a 3T Siemens scanner with a 32-channel head coil. Anatomical images (magnetization-prepared rapid acquisition with gradient echo (MPRAGE)) were first collected (repetition time = 2530ms, echo time = 1.74ms, flip angle = 7°, field of view = 256, voxel size = 1x1x1 mm^3^, 176 volumes).

The rs-fMRI was collected with patients fixating their gaze, instructed to stay awake and not think about anything in particular, using an echo-planar imaging sequence (repetition time = 3000ms, echo time = 30ms, flip angle = 85°, field of view = 72, voxel size = 3x3x3 mm^3^, 160 volumes, 8 minute acquisition).

During t-fMRI, patients were asked to decide whether words presented on a screen were abstract (i.e. a concept or idea) or concrete (i.e. something that can be touched). They responded by pressing a button with both thumbs, either using the left for abstract or right for concrete. This semantic decision task induces memory-encoding, and has proven relevance for the prediction of surgical memory outcome in TLE in terms of activation patterns [19]. Three runs of this sequence (repetition time = 2000ms, echo time = 20ms, flip angle = 90°, field of view = 64, voxel size = 3x3x3 mm^3^, 116 volumes and 3 minute 52 second acquisition per run) were collected.

### MRI analysis

**Fig 1:**
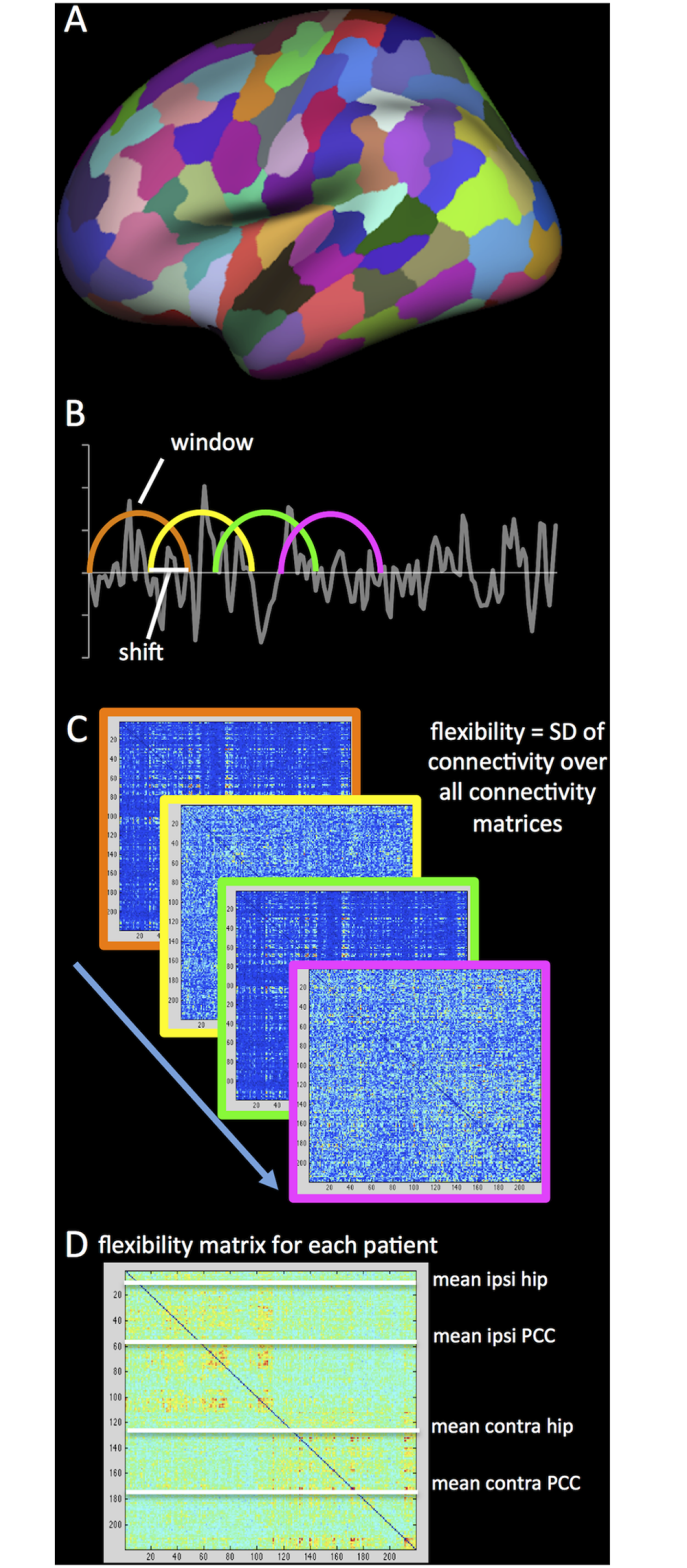
Schematic depiction of the analysis. The first step included parcellating the cortical surface into 219 parcels (A) and coregistering the rs-fMRI and t-fMRI to the anatomical scan. After preprocessing, sliding windows were determined in the entire scan (B). A connectivity matrix was calculated for each window, using Pearson correlation coefficients, after which the standard deviation (SD) of each connection over all windows was calculated to form a single flexibility matrix per patient (C). Finally, flexibility of the four regions-of-interest (ROIs: ipsilateral and cHC and PCC) with all other regions was averaged to obtain a single value for each ROI for both rs-fMRI and t-fMRI.

The analysis pipeline is schematically depicted in Fig 1. The MPRAGE volumes were used to reconstruct cortical surfaces using FreeSurfer version 4.5.0 [25,26]. The Desikan Atlas was used for cortical parcellation [27], which was later subdivided as described below. FreeSurfer was then used to co-register fMRI data to the reconstructed anatomical images. All included patients had less than 3mm absolute head movement during data collection, and less than 2 individual frame to frame movements larger than 0.5mm.

The rs-fMRI data were preprocessed using previously described techniques, namely (1) discarding the first four volumes of each run to ascertain T1-equilibrium, (2) slice timing correction, (3) head motion correction with FSL [28], (4) removal of constant offset and linear trend per run, (5) low-pass filtering below 0.08 Hz, (6) regressing out of six motion parameters, average signal of the whole brain, ventricles, and white matter [29]. Participants’ individual functional images were projected onto a template cortical surface, after which a 6mm full-width half-maximum (FWHM) smoothing kernel was applied and data were downsampled to 4mm resolution. The Lausanne 2008 parcellation scheme generated by the Connectome Mapper was used for subsequent atlas-based analysis of 219 cortical surface parcels [30,31]. Average time series were extracted from each of the parcels, by averaging time series of all voxels per parcel.

For t-fMRI, a comparable analysis was performed to assess connectivity and flexibility. The first four volumes of each run were removed, slice timing and motion were corrected, offset and trend were removed, data were low-pass filtered below 0.08 Hz, and motion, ventricles, white matter, and global signal were regressed out (all identical to rs-fMRI pre-processing). A 6mm FWHM kernel was applied and data were downsampled to 4mm, after which time series were extracted from the abovementioned 219 cortical surface parcels. From the 116 volumes, only those that occurred during semantic decision-making (i.e. 52 volumes) were used, while contrast volumes (cross hair fixation) were discarded. The three runs were temporally concatenated for subsequent flexibility analysis over the resulting 156 volumes.

### Flexibility calculation

For all subsequent analyses, we defined four regions of interest (ROIs), namely the ipsilateral (i.e. left) and contralateral (i.e. right) hippocampus and PCC. Custom-made Matlab (Matlab r2012a, Mathworks, Inc) scripts were used to perform flexibility analysis of the hippocampi and PCCs. A sliding window approach (102s windows, 12s shift, 30 windows total in rs-fMRI and 17 windows total in t-fMRI) was used. Subsequently, Fisher-transformed Pearson’s correlation coefficients of the four ROIs with all other 218 parcels were calculated for each window. Window length adhered to previous literature investigating connectivity dynamics in epilepsy patients [8], while using a reasonable number of time points for correlation calculation (34 for rs-fMRI and 51 for t-fMRI). With respect to shift, a recent study in healthy control subjects reports little effect of shift length on outcome [32].

Flexibility scores of the ROIs were determined by calculating the standard deviation of all 218 connections of each ROI to the rest of the brain over all windows, a method similar to that used for task-based flexibility in [33].

ROI flexibility was defined as the average of these 218 values for each ROI (both contralateral and ipsilateral to the epileptic focus), for both rs-fMRI and t-fMRI. Stationary connectivity was also computed, by calculating average absolute Fisher-transformed correlation coefficients of the four ROIs with the rest of the brain over the entire length of the scan after identical preprocessing.

### Statistical analysis

Statistical analysis was performed with PASW Statistics version 20. Because of the small sample size and non-Gaussian distribution of the data, non-parametric tests were used. Any differences between patients with or without memory disturbance using Mann-Whitney tests, while correcting for multiple comparison for each of the four ROIs using the Bonferroni correction, setting the level of significance to p < 0.0125. Correlations between flexibility indices and other variables were tested by means of Kendall’s Tau coefficients.

## Results

### Patient characteristics

In total, 21 LTLE patients were eligible, 5 of which moved excessively during functional scans (we used the threshold of 3mm movement on average or more than two movements greater than 0.5mm between time points). The remaining 16 patients formed our final sample (see Table 1 for patient characteristics). Ten patients scored below the 5^th^ percentile on one or more memory subtests, while six had preserved memory functioning according to our operationalization (see Table 2 for all memory subtest percentile scores). These two groups did not differ regarding any of the clinical and imaging variables reported in Table 1 (all p > 0.05). Full-scale IQ was measured at the time of memory testing, and was available in 11 of 16 patients. Full-scale IQ, which is confounded by disease-related processes, was significantly lower in memory disturbed patients (see Table 3). There was no measure of premorbid IQ.

**Table 1:** Patient characteristics.

	age	sex	mem dich	mem wei	onset	dur	freq	lesion	L hip	R hip	AEDs
1	29	F	D	0.9	21	8	1	MTS	3652	3601	ZNS,OCBZ
2	52	M	D	0.8	11	41	60	MTS	4439	4219	CBZ
3	41	F	D	0.86	27	14	10	none	4381	3534	LEV,PHT
4	25	M	P	1	13	12	1	none	4176	4435	LEV,LTG
5	58	F	P	1	1	57	30	MTS	2679	3773	LEV,LTG
6	20	M	P	1	16	4	10	MTS	4776	5013	OCBZ,LEV
7	35	F	P	1	10	25	45	none	4266	4042	LTG,RFM
8	32	M	P	1	27	5	8	none	4684	5075	LEV,LTG,OCBZ
9	30	M	D	0.9	16	14	U	MTS	3809	4936	CBZ.LEV
10	26	M	D	0.75	10	16	14	none	4622	4197	LTG,ZNS
11	46	M	D	0.5	33	13	U	none	4054	4125	TPM,FLB
12	59	M	D	0	15	44	3	MTS	2353	4399	TPM,LTG
13	50	F	D	0.8	40	10	1	none	3277	3394	LEV,LTG
14	59	F	D	0.2	8	51	U	MTS	2272	3367	U
15	41	F	D	0.6	31	10	0	MTS	4137	4040	LEV,LCM
16	47	M	P	1	39	8	1	none	4363	4157	CBZ

Note. Lat = lateralization of temporal lobe epilepsy, mem dich = dichotomized memory disturbance score, mem wei = number of tests < 5^th^ percentile / total number of administered tests, onset = age of onset, dur = duration of disease, freq = monthly seizure frequency, L hip = left hippocampal volume, R hip = right hippocampcal volume, AEDs = anti-epileptic drugs, U = unknown, R = right, L = left, MTS = mesial temporal sclerosis, CBZ = carbamazepine, CLZ = clonazepam, ZNS = zonisamide, OCBZ = oxcarbazepine, LEV = levetiracetam, PHT = phenytoin, LTG = lamotrigine, RFM = rufinamide, TPM = topiramate, FLB = felbamate, VPA = valproic acid, LCM = lacosamide.

**Table 2:** Memory subtest performance and memory disturbance score per patient.

Patient	1	2	3	4	5	6	7	8	9	10	11	12	13	14	15	16
SRT immediate	30	14	7			6	21		2	23	1			1		95
SRT delayed	67	50				42	50		50	50	17			0		75
RAVLT immediate				50	7			16				1	8		25	
RAVLT delayed																
7/24 immediate	99	1	5			63	99		45	3	73			1		45
7/24 delayed	79	2	37			18	79		79	18	58			7		79
BVMT immediate				50	27			76				1	12		1	
BVMT delayed				84	73			73				1	1		1	
WMS LM I	37	16	63		11		37		75			5	75	16	16	37
WMS LM II	75	25	50		16		37		75			1	50	2	25	9
WMS PA I	50	50	16				37		16		1			1		50
WMS PA II	84	50	25				63		25		5			5		50
WMS VR I	1	50	7				9		91					2		84
WMS VR II	16	37	8				9		84					2		84
Memory dist dich	D	D	D	P	P	P	P	P	D	D	D	D	D	D	D	P

Note. SRT = selective reminding test, RAVLT = Rey auditory verbal learning test, BVMT = brief visuospatial memory test, WMS = Wechsler memory scale, LM = logical memory, PA = paired associates, VR = visual reproduction, dist dich = disturbance score dichotomized, D = disturbed, P = preserved. Scores indicate percentile scores for each subtest, compared to normative data.

**Table 3:** Characteristics of patients with preserved and disturbed memory functioning.

Variable	Preserved memory (n = 6)	Disturbed memory (n = 10)
Age (years)	32.3 (13.7)	43.3 (12.1)
Males (female)	3 (3)	5 (5)
Left TLE (RTLE)	NA	NA
Lesion: none (MTS)	3 (3)	4 (6)
Age of onset (years)	14.3 (8.8)	21.2 (11.0)
Duration of disease (years)	18.0 (20.7)	22.1 (16.4)
Monthly seizure frequency	15.8 (17.9)	12.7 (21.5)
Full scale IQ (available in 5 and 6 patients)*	106 (10)	88 (11)
iHC volume (mm3)	4194 (779)	3700 (832)
cHC volume (mm3)	4466 (516)	3981 (503)
Average motion during rsfMRI (mm)	0.147 (0.1)	0.123 (0.07)
rs-fMRI iHC connectivity	0.024 (0.078)	0.033 (0.078)
rs-fMRI cHC connectivity	0.039 (0.104)	0.026 (0.065)
rs-fMRI iPCC connectivity	0.109 (0.056)	0.058 (0.098)
rs-fMRI cPCC connectivity	0.106 (0.056)	0.065 (0.088)
t-fMRI iHC flexibility	0.132 (0.037)	0.130 (0.033)
t-fMRI cHC flexibility	0.141 (0.024)	0.132 (0.033)
t-fMRI iPCC flexibility	0.150 (0.050)	0.129 (0.026)
t-fMRI cPCC flexibility	0.152 (0.047)	0.127 (0.024)
t-fMRI iHC connectivity	0.043 (0.032)	0.040 (0.041)
t-fMRI cHC connectivity	0.011 (0.037)	0.041 (0.049)
t-fMRI iPCC connectivity	0.009 (0.021)	0.001 (0.043)
t-fMRI cPCC connectivity	0.006 (0.025)	0.005 (0.025)

Note. PCC = posterior cingulate cortex, rs-fMRI = resting-state fMRI, t-fMRI = task fMRI. Elements indicate mean (standard deviation). There were no significant differences in these variables between patients with preserved versus disturbed memory functioning, apart from full IQ which was significantly lower in memory disturbed patients (* p = 0.030).

### Stationary connectivity not associated with memory disturbance

Stationary connectivity of the ipsilateral and contralateral hippocampus (iHC and cHC, respectively.) and ipsilateral and contralateral PCC (iPCC and cPCC, respectively) with the rest of the brain, averaged over the whole rs-fMRI scan, was also not different between patients with and without memory disturbance (see Table 2; iHC p = 0.958, cHC p = 0.635, iPCC p = 0.147, cPCC p = 0.181), and neither was stationary connectivity during t-fMRI (p = 0.562, p = 0.313, p = 0.999, p = 0.999, respectively).

### Resting-state flexibility, not task-based flexibility, related to memory disturbance

**Fig 2:**
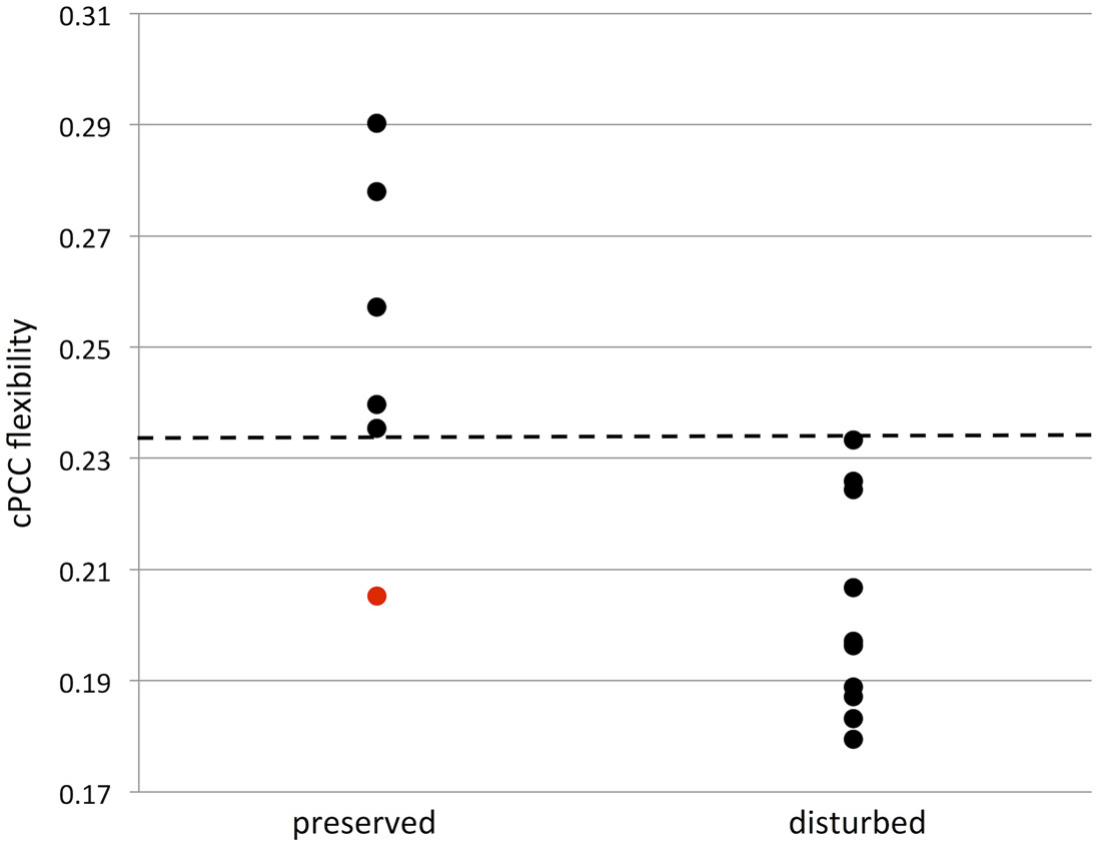
Memory and cPCC flexibility. Scatterplot of contralateral posterior cingulate cortex (cPCC) flexibility and memory. Patients with memory disturbance had decreased cPCC flexibility compared to memory preserved patients (p < 0.01). Dotted line indicates a threshold of 0.234, which separates memory preserved and disturbed patients, apart from patient 8 in Table 1 (indicated in red in this figure).

Hippocampal flexibility did not differ between patients with and without memory disturbance (iHC p = 0.958, contralateral p = 0.635). In contrast, cPCC flexibility was significantly lower in patients with disturbed memory compared to patients with preserved memory functioning after correction for multiple comparisons (Fig 2; p = 0.003), while the same result was trending in the iPCC (p = 0.093). Flexibility was significantly correlated between the iPCC and cPCC (Tau = 0.550, p = 0.003), further pointing towards a bilateral pattern, instead of an effect specific to the contralateral PCC.

Flexibility during t-fMRI was not significantly different between the two groups (see Table 3; iHC p = 0.713, cHC p = 0.492, iPCC p = 0.263, cPCC p = 0.368). Furthermore, resting-state PCC flexibility was not significantly related to task-based PCC flexibility (iPCC Tau = -0.067, p = 0.718, cPCC Tau = 0.200, p = 0.280).

Memory disturbed LTLE patients displayed 22% less cPCC flexibility (p = 0.003) and 14% less iPCC flexibility (p = 0.093) compared to memory preserved LTLE patients. When looking only within each patient’s own network, the memory disturbed LTLE patients’ cPCC had on average 10% less flexibility than their individual average over all brain regions, while memory preserved LTLE patients showed 4% higher than average flexibility for the cPCC (p = 0.011). These percentages came to 4% and 2% for the iPCC (p = 0.562). These results indicate a flexibility shift of the PCC in memory disturbed LTLE patients, not only at the group level, but also within the individual brain network.

**Fig 3:**
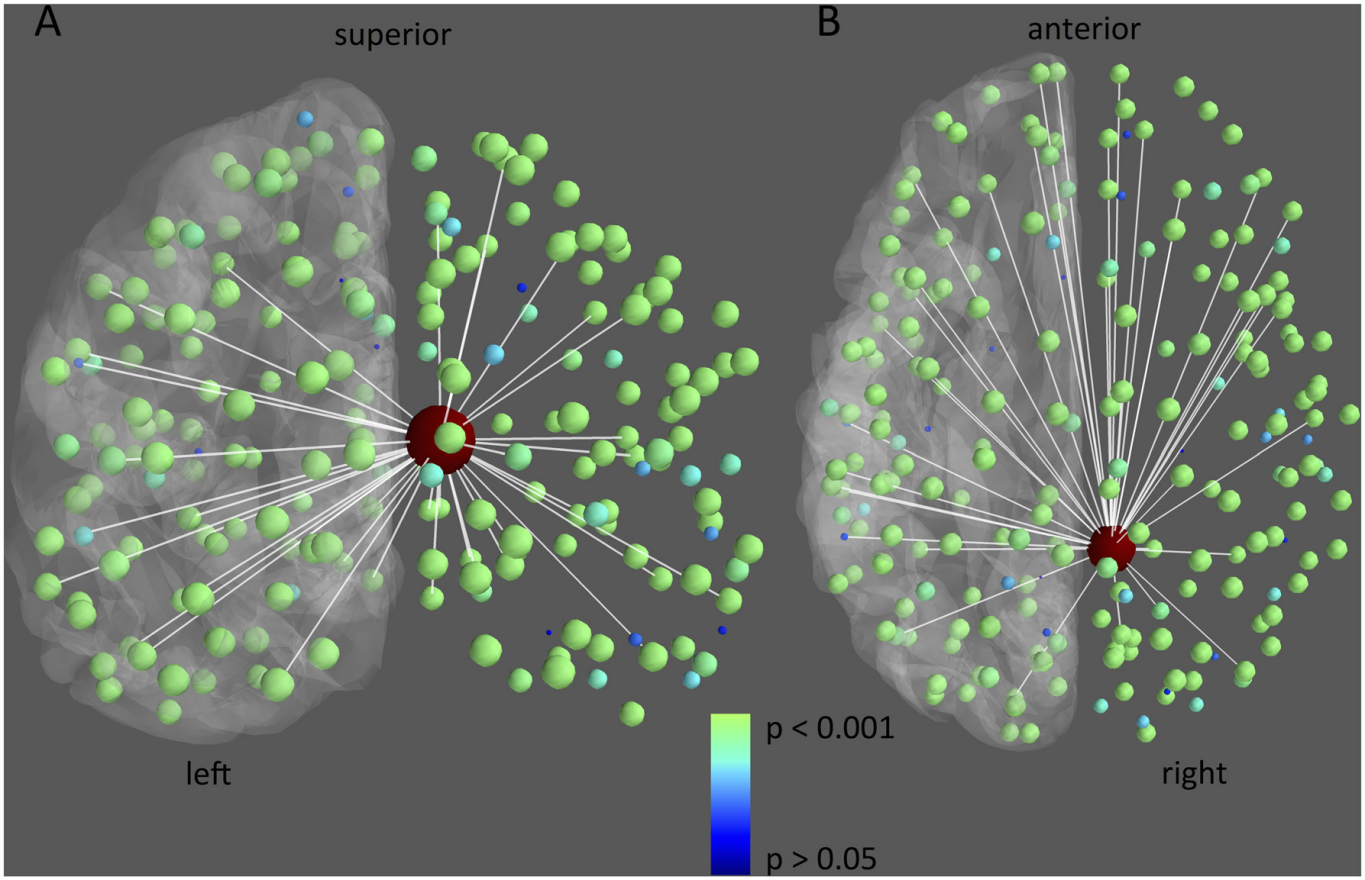
Differences in resting-state flexibility of connections from the cPCC between memory preserved and disturbed patients. The 5% largest differences in connections between memory preserved and memory disturbed LTLE patients (i.e. preserved—disturbed) are depicted in white. Furthermore, nodal group differences in flexibility with the cPCC (large node in red) are depicted in color and size, with smaller blue nodes indicating no significant difference (P > 0.05), and bigger green nodes referring to significant decreases in resting-state flexibility in the memory disturbed patients (P < 0.001). For reference, the left cortical surface is displayed in grey.

Moreover, memory disturbed and preserved LTLE patients were separated with respect to cPCC flexibility. Using a cPCC flexibility threshold of 0.234, all but one LTLE patient could be classified correctly (accuracy = 94%). When investigating the spatial distribution of the difference in flexibility between memory preserved and disturbed LTLE patients (Fig 3), it becomes clear that PCC connections are implicated throughout the brain, instead of being focused on connections to any particular regions of the brain.

Memory disturbed patients had lower IQ at the time of testing than preserved patients. However, cPCC flexibility was not significantly related to full-scale IQ (Kendall’s Tau = 0.220, p = 0.349), indicating that although memory disturbance and lower IQ may be due to the same cognitive phenotype, cPCC flexibility seems to be specifically related to memory functioning in these patients. Furthermore, average relative motion was also not significantly related to cPCC flexibility (Kendall’s Tau = -0.067, p = 0.719), indicating that this was also not likely to be a confounding factor. Finally, the consistency of our findings across multiple window lengths was investigated (see Table 4), showing identical results.

**Table 4:** cPCC flexibility with varying window lengths in memory preserved and disturbed patients.

	Preserved (n = 6)	Disturbed (n = 10)	p-value
102s windows	0.251 (0.031)	0.202 (0.019)	0.003**
87s windows	0.247 (0.013)	0.221 (0.014)	0.003**
60s windows	0.304 (0.014)	0.280 (0.020)	0.016*

Note.

** p<0.05,

* p<0.01. Values indicate mean (SD) of cPCC flexibility. P-values based on Mann-Whitney U-tests.

## Discussion

We show, for the first time, associations between resting-state dynamic connectivity and cognitive functioning in epilepsy patients. Resting-state flexibility of the PCC was related to memory functioning in LTLE patients. Patients with memory disturbances had 22% less cPCC flexibility and 14% less iPCC flexibility than memory-preserved patients. Moreover, this dynamical measure of connectivity was a sensitive predictor of preserved versus disturbed memory functioning in LTLE, able to classify 94% of cases correctly. These results indicate that preserved flexibility of the PCC is relevant for adequate memory functioning in the setting of LTLE.

Several studies have investigated dynamic connectivity during task performance, particularly during working memory and learning tasks [15,17,33]. These studies indicate that higher variability of connectivity is beneficial for performance. One study has reported on the association between resting-state dynamic connectivity and cognitive performance [18]. Indeed, flexibility during the resting-state proved to explain more behavioral variance than stationary connectivity as is the case in our current results. Having increased dynamics within the resting-state was associated with better cognitive performance. Taken together with our current results, flexible connectivity at rest seems to be a beneficial feature of the brain network. Speculatively, higher variability of connectivity may benefit information processing within the connectome, as has been postulated in other types of complex networks as well as the brain [34]. Having a flexible PCC may prohibit overuse of this important hub in the brain network. More research is necessary to investigate the precise mechanisms underlying the association between flexibility and cognition, particularly in healthy subjects.

Stationary connectivity, i.e. average connectivity strength over the entire length of the rs-fMRI scan, did not relate to memory functioning in our sample. Previous literature on this stationary measure seems to converge towards better memory functioning being related to decreased ipsilateral and/or increased contralateral connectivity of the hippocampus and PCC [9–12]. Our results suggest that flexibility of posterior DMN areas may be a more sensitive measure of cognitive functioning than stationary connectivity in this patient population.

In terms of spatial specificity, the loss of flexibility was spread over many connections between the PCC and the rest of the brain. This flexibility correlate of cognition fits with the well-described vision of the PCC as a connector hub between different functional subsystems [35–37]. In terms of memory, the DMN encompasses multiple memory-related areas, with the PCC possibly forming the ‘gate-keeper’ of memory traces in the brain [38]. Reduced flexibility of this area in patients with disturbed memory function suggests that the dynamics of the PCC are indeed important for adequate encoding and retrieval.

The specificity of our results for LTLE memory functioning, however, is limited. Some patients had classical verbal memory deficits, while others showed visual memory impairments. Moreover, memory score was also related to overall IQ measured at the time of memory testing, indicating that preserved and disturbed memory functioning in this particular study population may be indicative of a generally different cognitive phenotype. Overall IQ is confounded by disease processes and thus does not indicate pre-existent differences in intelligence between the groups, but we unfortunately did not have a measure of premorbid IQ. Future studies with larger sample size and more extensive neuropsychological testing are needed to investigate the specificity of flexibility for different cognitive domains. In our sample, full-scale IQ was not related to cPCC flexibility. Therefore, this study suggests that specifically clinical measurements of memory functioning outside the scanner are related to resting-state PCC flexibility.

We did not find any group differences between flexibility or stationary connectivity during a semantic decision task related to memory outcome after surgical intervention. Previous studies have related increased flexibility to better performance during scanning [15,37], but there is no previous literature linking task-based flexibility to cognitive performance. A possible confounder in this lack of association could be limited signal-to-noise ratio in the t-fMRI data, as these runs had lower TR and shorter total scanning time. This means that there were fewer windows over which to calculate flexibility, which may have impacted statistical power to detect associations.

Several open questions remain regarding our results. The optimal method of assessing the dynamics of connectivity has not been determined [13]. In the current study, we use a relatively simple and straightforward method of determining flexibility, comparable to a recent study [33]. Furthermore, confounding effects of physiological noise, motion, and length of the sliding windows may theoretically influence results when performing dynamic connectivity analysis. However, our study design (i.e. within patient groups with presumed similar confounding parameters) should mitigate most of these confounders. Additionally, our analysis was limited to PCC and hippocampal flexibility alone, as these areas have most extensively been reported to differ in TLE when using stationary connectivity, and are thus provided with an excellent context for determining the added value of dynamical connectivity. Furthermore, the influence of motion and choice of window lengths on our results proved to be non-significant.

An interesting avenue for further investigation is whether decreased flexibility of the PCC in memory-compromised LTLE patients signifies that a decreased number of network topologies is alternated during the resting-state, as found in schizophrenia patients [16], although more data points would be necessary for such analysis. Also, dynamical connectivity has been shown in anesthetized monkeys [39], suggesting that fluctuating connectivity is not only relevant for higher cognitive functioning as investigated in the current study, but also forms a basic neuronal principle. Finally, it would be interesting to relate t-fMRI flexibility with performance score on the memory-encoding task.
