# Correction: Loss of Resting-State Posterior Cingulate Flexibility Is Associated with Memory Disturbance in Left Temporal Lobe Epilepsy

**DOI:** 10.1371/journal.pone.0148664

**Published:** 2016-02-01

**Authors:** Linda Douw, Catherine L. Leveroni, Naoaki Tanaka, Britt C. Emerton, Andrew J. Cole, Claus Reinsberger, Steven M. Stufflebeam

The fifth author’s name is spelled incorrectly. The correct name is: Andrew J. Cole. The correct citation is: Douw L, Leveroni CL, Tanaka N, Emerton BC, Cole AJ, Reinsberger C, et al. (2015) Loss of Resting-State Posterior Cingulate Flexibility Is Associated with Memory Disturbance in Left Temporal Lobe Epilepsy. PLoS ONE 10(6): e0131209. doi:10.1371/journal.pone.0131209
